# Reaction Steps in Heterogeneous Photocatalytic Oxidation of Toluene in Gas Phase—A Review

**DOI:** 10.3390/molecules28186451

**Published:** 2023-09-06

**Authors:** Yerzhigit Tulebekov, Zhandos Orazov, Bagdat Satybaldiyev, Daniel D. Snow, Raphaël Schneider, Bolat Uralbekov

**Affiliations:** 1 Center of Physical-Chemical Methods of Research and Analysis, Al-Farabi Kazakh National University, Almaty 050012, Kazakhstan; yerjigit@gmail.com (Y.T.); zhandosorazov98@gmail.com (Z.O.); bagdat.satybaldiev@gmail.com (B.S.); 2 LLP «EcoRadSM», Almaty 050040, Kazakhstan; 3 Water Sciences Laboratory, Nebraska Water Center, Part of the Daugherty Water for Food Global Institute, University of Nebraska, Lincoln, NE 68583, USA; dsnow1@unl.edu; 4 Université de Lorraine, CNRS, LRGP, F-54000 Nancy, France; raphael.schneider@univ-lorraine.fr

**Keywords:** photocatalytic oxidation, toluene, titanium dioxide, reaction pathways

## Abstract

A review of the current literature shows there is no clear consensus regarding the reaction mechanisms of air-borne aromatic compounds such as toluene by photocatalytic oxidation. Potential oxidation reactions over TiO_2_ or TiO_2_-based catalysts under ultraviolet and visible (UV/VIS) illumination are most commonly considered for removal of these pollutants. Along the pathways from a model pollutant, toluene, to final mineralization products (CO_2_ and H_2_O), the formation of several intermediates via specific reactions include parallel oxidation reactions and formation of less-reactive intermediates on the TiO_2_ surface. The latter may occupy active adsorption sites and causes drastic catalyst deactivation in some cases. Major hazardous gas-phase intermediates are benzene and formaldehyde, classified by the International Agency for Research on Cancer (IARC) as Group 1 carcinogenic compounds. Adsorbed intermediates leading to catalyst deactivation are benzaldehyde, benzoic acid, and cresols. The three most typical pathways of toluene photocatalytic oxidation are reviewed: methyl group oxidation, aromatic ring oxidation, and aromatic ring opening.

## 1. Introduction

Air pollution with aromatic compounds remains a dangerous human health problem in urban and industrial areas, and photocatalytic oxidation is a widely promoted air purification technology for these substances. One of the significant limitations of photocatalytic technology for air purification is the partial oxidation of organics on the catalyst surface, which may lead to the formation of more toxic by-products in the gas phase than the original pollutants. Many studies have shown that, in addition to full mineralization of organic compounds to the final products (H_2_O and CO_2_) during photocatalytic oxidation (PCO) of organic pollutants, the formation of less-reactive and often more strongly adsorbed intermediates is a likely process [1,2,3,4]. While considerable effort has been paid to study organic pollutants, many questions remain regarding the conditions required for formation of stable oxidation by-products, the mechanisms of photocatalytic decomposition, the effect of water, and other factors on oxidation rate and reaction mechanisms.

Quite a number of reviews on photocatalytic oxidation of VOCs have been published; however, most of these reviews are devoted to improving catalyst performance, the effect of extensive/intensive reaction conditions, and reactor variables, and few reviews have paid attention to by-products’ toxicity and BTEX degradation mechanisms [1,2,3,5,6,7]. Herein, we attempt to give an overview of toluene photocatalytic oxidation over TiO_2_ with a focus on photocatalytic oxidation by-products, reaction mechanisms, and risk assessment issues. TiO_2_ or TiO_2_-based materials are widely investigated as photocatalysts, while toluene is the most extensively studied as a model aromatic in the photocatalytic oxidation research. Chemical stability, cost-effectiveness in preparation, and non-toxicity make TiO_2_ attractive for use in photocatalysis; in addition, its ability to operate at room temperature and its relatively high decomposition rate of pollutants should be separately mentioned [3,5,6,8].

Aromatic compounds like benzene, toluene, ethylbenzene, and xylene (BTEX) are polluting volatile organic compounds (VOCs) widespread in the atmosphere of urban and industrial areas [9,10,11]. This group of compounds has recently been of particular concern due to their impact on the environment, formation of photochemical smog, and human health issues from chronic exposure, which include various respiratory diseases and cancerogenic effects [5,9,12,13]. Emissions from the combustion of gasoline and diesel fuels are the largest contributors to atmospheric BTEX concentrations [9]. Regarding toluene, which is the least toxic of the BTEX group, it is the most abundant aromatic type in the atmosphere [14,15]. Toluene is considered as the VOC with the highest contribution to ozone formation, owing to a combination of high emission and a relatively high photochemical ozone-creation potential [16,17]. The French normalization group recommended using toluene as model pollutant for evaluation of photocatalytic system performance [18].

## 2. Basic Principles of Photocatalysis

PCO requires the excitation of an electron from the valence band to the conduction band, which proceeds when the energy of the absorbed photon exceeds the band gap of the semiconductor material [3,19,20,21,22]. The semiconductor used for PCO must have a suitable band gap that will match the energy of the light source used to excite electrons from the valence band to the conduction band, generating electron–hole pairs, which in turn are required for photocatalytic reactions. The value of E_g_ of semiconductor photocatalysts determines the energy required to excite an electron from the valence band to the conduction band by absorbing light. For efficient photocatalysis, the semiconductor should have a sufficiently narrow band gap (1.23 eV < E_g_ < 3.0 eV) so that the electron excitation energy is correspondingly low [23]. The band gap E_g_ for the widely used anatase form of TiO_2_ is 3.2 eV, in which the energy potentials of the valence band and the conduction band are 0.37 V (vs. NHE) and +2.83 V (vs. NHE), respectively [24]. The smaller the E_g_ value, the wider the spectral range of the light response, resulting in efficient use of visible light. The spectral range of the light response for semiconductors can be enlarged by doping or co-doping with metals and nonmetals or by preparing heterostructures, which will further narrow the band gap, e.g., (N, Ta)-TiO_2_, C-TiO_2_, g-CN/TiO_2_, and ZnO/TiO_2_/RGO [24,25,26,27,28,29,30,31].

The founders of the photodecomposition process and later the originators of the term “photocatalysis” are Honda and Fujishima. Their work demonstrated that TiO_2_ can be used as a catalyst for the decomposition of water into hydrogen and oxygen in the presence of sunlight as an energy source, which is a potentially important step towards clean and renewable energy [32]. Since then, many researchers have developed various photocatalytic materials and processes for energy applications, taking into account several criteria: band gap, chemical stability, carrier mobility, surface area, cost and availability, and redox potential.

One of the main criteria for efficient photocatalytic activity is carrier mobility. A semiconductor with high carrier mobility can provide efficient transport of electrons and holes to the reaction sites. This minimizes recombination and increases the efficiency of the photocatalytic process [33]. Recombination decreases the lifetime of charge carriers and limits their oxidizing/reducing properties on the surface of the semiconductor. However, the recombination process is dependent on defects, doping, and the formation of hetero-structured photocatalysts. Thus, in order to increase the lifetime of charge carriers, the use of high-quality crystals is required [34].

In general, a semiconductor that meets the above criteria is the most efficient for photocatalytic processes. The most common photocatalysts are semiconductors such as titanium dioxide, zinc oxide, and cadmium sulfide, which can absorb photons and generate the electron–hole pairs involved in redox reactions. These semiconductors have been extensively studied and have shown high photocatalytic activity in a variety of reactions including hydrogen production, water splitting, and pollutant degradation [32,35,36].

## 3. General Issues in Gaseous Organic Compounds Photocatalytic Oxidation

Photocatalytic oxidation of gaseous organics includes several processes, the foremost of which include the formation and recombination of electrons and holes, the adsorption of pollutants and chemical reactions, and the desorption of products [3]. The initial stage of photocatalytic oxidation is the generation of electron–hole pairs under illumination, which can then migrate to the catalyst surface and are trapped at different sites. Specifically, photogenerated electrons can further reduce adsorbed molecules on the catalyst surface, such as O_2_ or organic molecules. On the other hand, photogenerated holes can oxidize water molecules to form reactive oxygen species like hydroxyl radicals (OH•) and hydrogen peroxide as well as oxide the organic pollutants.

Electron–hole pairs can recombine instead of participating in surface reactions [37], which reduces the efficiency of the photocatalytic reaction. It is important to prevent recombination by improving the separation of electrons and holes via doping, precipitation of cocatalysts, or via “hetero-structuration”. Using nanomaterials can greatly improve how electrons reach the surface of the catalyst, thus reacting with electron acceptors in a shorter transfer distance [3]. 

**Figure 1:**
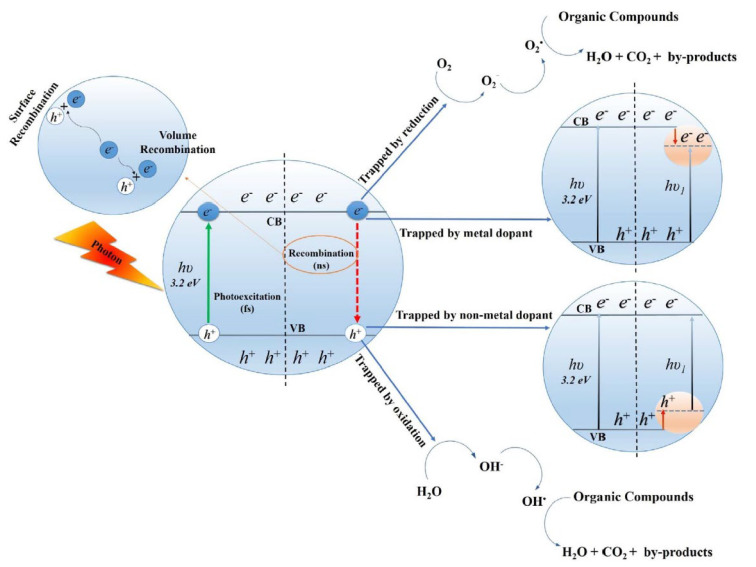
Basic principle of PCO for removal of VOCs. (Reprinted from Shayegan et al. [3], 2018, with permission from Elsevier).

The mechanism of the photocatalytic oxidation of gaseous organic substances, summarized in Figure 1, and the reaction steps can be represented as follows:

1. Charge-carrier generation.

Absorption of a light quantum equal to or greater than the band gap of a semiconductor and formation of charge carriers, i.e., electrons (e^−^) and holes (h^+^), can be generated in femtoseconds [3]:

(1) $${\mathrm{TiO}}_{2}+\mathrm{hv}\to h^{+}+e^{-}$$

2. Charge-carrier recombination. The photogenerated electrons can recombine in the range from microseconds to nanoseconds, and holes can recombine rapidly in a few nanoseconds [21]:

(2) $$h^{+}+e^{-}\to \mathrm{heat}\left(\mathrm{phonons}\right)\;\left(\mathrm{electron–hole recombination}\right)$$

3. Generation of reactive oxygen species:

Hydroxyl radicals are produced by dissociative chemisorption of water on the catalyst surface followed by the oxidation of adsorbed OH^−^ [21]:

(3) $$H_{2}O\to {\mathrm{OH}}^{-}+H^{+}$$

(4) $${\mathrm{OH}}^{-}+h^{+}\to \mathrm{OH}•$$

Superoxide (O_2_^•−^) and hydroperoxyl radicals (HO_2_^•^) are generated through reduction of adsorbed O_2_ molecules [38]:

(5) $$O_{2}+e^{-}\to O_{2}{}^{-•}$$

(6) $$O_{2}{}^{-•}+H^{+}\to {\mathrm{HO}}_{2}•$$

The HO_2_• radical is an electron acceptor that may produce hydrogen peroxide (H_2_O_2_). H_2_O_2_ as an oxidizing agent and decreases the recombination rate of electrons and holes by capturing photo-induced electrons, thereby enhancing PCO efficiency [3]. The produced H_2_O_2_ oxidizes adsorbed oxygen and reacts with available charge carriers [39] but may also react with a photon, which leads to the formation of OH• radicals [40]:

(7) $${\mathrm{HO}}_{2}•+e^{-}\to {\mathrm{HO}}_{2}{}^{-}\left(\mathrm{electron trapping}\right)$$

(8) $${\mathrm{HO}}_{2}{}^{-}+H^{+}\to H_{2}O_{2}$$

(9) $$H_{2}O_{2}+O_{2}{}^{-•}\to \mathrm{OH}•+{\mathrm{OH}}^{-}+O_{2}$$

(10) $$H_{2}O_{2}+e^{-}\to \mathrm{OH}•+{\mathrm{OH}}^{-}$$

(11) $$H_{2}O_{2}+\mathrm{hv}\to 2\mathrm{OH}•$$

## 4. Adsorption of BTEX to TiO_2_ Surface

It has been shown that adsorption of benzene and toluene on rutile (TiO_2_) surfaces depends on the degree of surface hydroxylation [41]. Infrared (IR) spectra of adsorbed aromatic species and comparison with IR spectra of gaseous species showed that formation of a Ti^4+^⋯π–electron type complex is most likely for dehydroxylated surface, while formation of an OH⋯π–electron type complex is observed for hydroxylated surface. Furthermore, the area occupied by an adsorbed molecule leads to an assumption that these aromatic molecules are adsorbed in a flat orientation with relatively loose packing on the rutile surface. The presence of an electron-donating methyl group results in a stronger interaction of toluene with surface OH groups compared with benzene. Based on measured irreversible adsorption of benzene and toluene, the authors suggested that the strength of the charge-transfer interaction is in the order Ti^4+^···π–electrons > OH···π–electrons [41]. This mechanism of interaction between OH groups and the π–electrons of the toluene was observed [42] while studying toluene adsorption by Fourier-transform IR spectroscopy (FT-IR). The authors also showed that this interaction between aromatic molecules and the hydroxyl group is quite weak, as evidenced by restoring of OH bands after short outgassing of the sample.

Recent density functional theory (DFT) calculations further revealed that benzene and toluene adsorb on the TiO_2_ surface by configuring the aromatic ring parallel to the adsorption site and interacts through the π–electrons of the aromatic rings [43]. Additionally, using functional DFT showed that more than 50% of the adsorption energy of the BTEX gas-phase adsorption on TiO_2_ comes from dispersion, and the rest is most likely due to charge-induced polarization of the molecular electron density rather than from charge transfer [44]. Factors like temperature, gas pressure, and surface area can affect the adsorption capacity of the catalyst, the detail of which can be found elsewhere [6,7]. Note here that although surface area affects the adsorption capacity, most studies showed that surface chemistry plays a more critical role in gas-phase PCO. It has been shown that the adsorbed amount of organic compounds agrees with the number of surface reactive species (e.g., OH groups) [6,45], which is consistent with the above-mentioned mechanisms of adsorption through the formation of π-type complexes.

The Langmuir–Hinshelwood (L-H) kinetic form combined with integral law analysis can be used to describe the toluene photo-oxidation rate [46]. The authors also established that for a toluene concentration below 160 mg/m^3^, the oxidation rate is approximately first-order, while in the range of 160–550 mg/m^3^, the oxidation rate is between zero and first-order. Obee and Brown showed that the photocatalytic oxidation of toluene is affected by humidity (presence of water vapor in the air) [47], while the process of adsorption of toluene and water on the surface is described by the Langmuir–Hinshelwood (L-H) equation:

(12) $$r=k_{0}\theta_{p}\theta_{w}$$

(13) $$\theta_{p}=\frac{K_{1}C_{p}}{1+K_{1}C_{p}+K_{2}C_{w}}$$

(14) $$\theta_{w}=\frac{K_{4}C_{p}}{1+K_{3}C_{p}+K_{4}C_{w}}$$

where r is the oxidation reaction rate; k_0_ is the constant of proportionality; K_1_, K_2_, K_3_, K_4_ are the Langmuir adsorption equilibrium constants (ratio of adsorption to desorption rates); C_p_, C_w_ are the gas-phase concentrations of the aromatic molecule and water vapor. The parameters θp, θ_w_ are fractions of pollutant and water sorbed at the surface and refer to competitive adsorption between aromatic and water molecules for the same adsorption site. Note that this is a general equation for bimolecular form: by setting K_1_ = K_3_, K_4_ = K_2_, the true bimolecular form can be obtained, while by setting K_4_ = ∞ (i.e., θ_w_ = 1), the unimolecular form can be obtained [47].

The data showed that at moderate to high humidity (above ca. 5000 ppmv) and low toluene level (0.5 ppmv), the oxidation rate of toluene is likely the result of competitive adsorption of water and toluene molecules on the available hydroxyl adsorption site [47]. In addition to competitive adsorption, the formation and reduction of hydroxyl radical population (adsorption sites—bonding to the hydroxyl) plays an important role at very low humidity levels and high contaminant levels as well as at high humidity levels and high contaminant levels. An important result of this work [42] is that the toluene oxidation rate is first-order for toluene concentrations below 1 ppmv.

The study described by Sleiman et al. conducted in typical indoor toluene concentrations (ppbv) established that the photocatalytic reaction occurs on the photocatalyst surfaces through the Langmuir–Hinshelwood (L-H) mechanism and not in the gas phase [48]. The following expression was used for the reaction rate in the plug-flow reactor:

(15) $$r=-u\frac{{\mathrm{dC}}_{p}}{\mathrm{dL}}={\frac{{\mathrm{kK}}_{\;}C_{p}}{1+K_{\;}C_{p}}}_{\;}$$

where, in addition to the parameters specified in the Equations (12)–(14), the following parameters appear: L is length of the photoreactor, and u is the gas velocity through the reactor. This expression can be used assuming that the mass transfer is not a limiting step and that the effect of the intermediate product is negligible. Sleiman et al. argued that OH• radicals are not the main active species during the PCO of toluene, based on observed limited influence of relative humidity (RH) on the toluene conversation [48]. Specifically, they observed the quasi-total conversion and mineralization of toluene in the absence of water vapor gas phase (0% RH).

## 5. General Observation of Aromatic Molecules PCO Experiments

Numerous studies involving BTEX PCO have shown conflicting results on photocatalytic oxidation and kinetics of by-products formation most probably due to the use of different types of photoreactors, including different conditions for conducting PCO: inlet gas composition, gas flow rate, relative humidity, light energy, and irradiance as well as the presence of competitive sorbates and many other parameters. Because of their properties, the use of different analytical techniques to measure reactants and transformation products is also a problem. Detection of trace amounts of gas-phase by-products was difficult during the first stages of the study of heterogeneous photocatalysis from 1995 to 2000 due to analytical methods with high detection limits, such as gas chromatography (GC) with flame ionization detector (FID) [49]. Subsequently, with the use of more sensitive methods like gas chromatography with mass spectrometry (GC-MS) and proton transfer reaction-mass spectrometry (PTR-MS), it was possible to detect trace amounts of by-products in the gas phase. In GC-MS, purge and trap (PT) adsorption tubes or solid-phase microextraction (SPME) can be used to preconcentrate the gas-phase by-products from the outlet gas, thus producing more accurate results at expected concentrations.

The multiple advantages of PCO experiments for characterizing surface-intermediate species should be mentioned. PCO usually operates at room temperature, so it can be easily interrupted with less perturbation of the surface than non-photocatalytic reactions [50]. PCO can be conducted in transient and continuous-flow modes for aromatics photocatalytic oxidation studies, while intermediates can be measured either in gas or solid phase (see Table 1 for details). Various extraction methods are used to characterize the adsorbed organic species on the catalysis surface usually following steady-state PCO. One of the features of conducting transient experiments is the possibility of separately studying the processes of adsorption, surface reaction, and desorption [51].

The first attempts to detect aromatic by-products in the transient experimental condition were carried out by Larson and Falconer, who showed formation of less-reactive intermediates than the original aromatics during PCO of benzene and toluene [52]. Studying transient reactions of a toluene monolayer, they observed that toluene reacts quickly to form strongly bound intermediates that react more slowly to generate CO_2_ and H_2_O. Although they were unable to reliably identify these strongly bound intermediates, they found using temperature-programmed oxidation (TPO) and desorption (TPD) that these compounds are more strongly bound to TiO_2_ than native aromatics. Thus, the catalyst surface is expected to become covered and potentially deactivated with these intermediates.

This work was further developed with the use of temperature-programmed hydrogenation (TPH), which showed that these intermediates have a ring structure with a methyl (or oxidized methyl) group [51]. TPH resulted in saturated intermediates, so they are more weakly held and desorbed or oxidized during temperature increases. They reported that benzaldehyde adsorbs in two forms on the TiO_2_ surface, and these two forms have different reactivities. Investigating TPD, TPH, and TPO spectra, they concluded that toluene is most likely oxidized via benzaldehyde, for which the aromatic ring is adsorbed parallel to the catalyst surface rather than adsorbing perpendicular to the surface. According to their study, benzaldehyde formation is not a rate-limiting step but quickly reacts to form strongly bound, less-reactive intermediates.

In the following sections, other by-products in the gas- and solid-phases compounds are reported during PCO of toluene. In our review, we present the names of organic compounds, experimental conditions, and other parameters exactly as they are given in the corresponding articles.

### 5.1. Gas-Phase By-Products

One of the first works identifying gas-phase by-products was conducted by Ibusuki and Takeuchi, who used a relatively high concentration of toluene PCO on TiO_2_ [53]. They showed the formation of a small amount of benzaldehyde in addition to CO_2_ during toluene PCO [53]. In their study, they found that the concentration of formed CO_2_ increased linearly with increasing relative humidity, while the benzaldehyde concentration decreased with increasing relative humidity (RH).

A later work used GC-FID and high-pressure liquid chromatography (HPLC) to measure benzaldehyde as a major by-product in the gas phase during PCO of a continuous flow of toluene over anatase TiO_2_ [42]. In their study, benzene, benzyl alcohol, and trace amounts of benzoic acid and phenol in gas phase were detected, and the proposed mechanism involved the further oxidation of benzaldehyde into benzoic acid followed by a photo-decarboxylation. They also reported that cleavage of the CH_3_ group and α carbon of toluene did not occur, as small mass species like CH_4_ and CH_3_OH were not detected. By increasing the reaction time, benzaldehyde was the only gas-phase product detected and did not appear to oxidize further, resulting in deactivation of catalyst [54]. In contrast, other authors using TPH showed that only small amounts of benzaldehyde were present on the catalyst surface following continuous-flow PCO of both humidified and dry toluene [50]. They concluded that benzaldehyde is not the cause of the TiO_2_ catalyst deactivation, as it oxidized quickly to form less-reactive intermediates. Most likely, the discrepancy between two studies may arise from different levels of inlet toluene concentrations (see Table 1). Despite contrary results, the most important result of their studies is that toluene reacted quickly to form benzaldehyde.

The availability of more sensitive methods like GC-MS and proton transfer reaction mass spectrometry (PTR-MS) make it possible to detect new by-products, including small fragments in the gas phase, with high accuracy. For example, in addition to the previously identified benzaldehyde and benzene, Sleiman et al. used an automated thermal desorption technique coupled to GC-MS to detect for the first time small carbonyl compounds during continuous-flow toluene PCO at the ppbv level [48]. The reaction intermediates formed differed according to the relative humidity (RH). In dry toluene PCO, traces of low-molecular-weight carbonyls (formaldehyde, methyl glyoxal, etc.) were detected, while in humidified air, hydroxylated intermediates such as cresols, benzyl alcohol, and phenol were obtained (see Table 1 for details).

**Figure 2:**
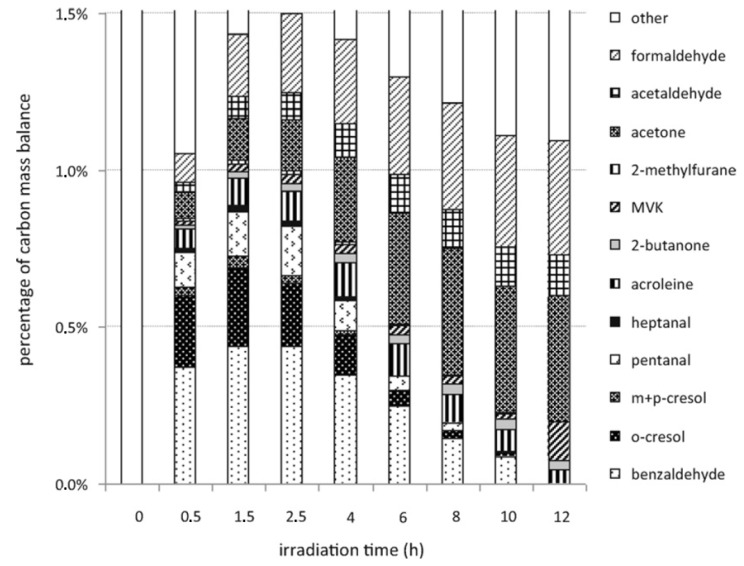
Contribution of the identified gaseous organic reaction intermediates to the reaction carbon mass balance during the photocatalytic degradation of 800 ppb of toluene in the presence of 13,000 ppm H_2_O, for various irradiation times. (Reprinted from Debono et al., 2011 [56], with permission from Elsevier.)

A further development in the PCO study of toluene by-products in the gas phase at ppbv level was provided by Mo et al., who used PTR-MS to detect by-products in continuous-flow PCO over a TiO_2_ catalyst [55]. In addition to already mentioned gas-phase by-products, the authors detected for the first time gaseous methanol, propylene, acetaldehyde acetone/propionaldehyde, and acetic acid. New intermediate species like acroleine, pentanal, heptanal, and 2-methylfurane were also identified during toluene PCO at the ppbv level in a static batch reactor [56]. More important for practical applications, the authors showed that contribution of the identified reaction intermediates in gas phase on the carbon balance ranged from 1 to 1.5% until 12 h of irradiation (see Figure 2). Figure 2 presents the changes of reaction intermediates with irradiation time, starting from the generation of aromatic compounds at initial stage through aliphatic-oxidized VOCs to formaldehyde, acetone, and acetaldehyde at the final stage of PCO.

**Table 1:** Gas-phase by-products identified during PCO over TiO_2_.

Catalyst/Method of Preparation	Experimental Conditions	Analytical Method	Identified Gaseous Products	Reference
Reagent grade TiO_2_ BET surface area: 10.4 m^2^/g	Inlet toluene conc. (ppm): 80 Flow rate (mL min^−1^): 300	GS-FID HPLC	Humidified and un-humidified condition: CO_2_, Benzaldehyde.	[53]
TiO_2_ in the anatase phase BET specific surface area 10 m^2^ g^−1^	Inlet toluene conc. (molar fraction): 4.0 × 10^−4^ to 1.3 × 10^−2^ Flow rate (cm^3^ s^−1^) 0.17–10 Reaction temperature (K): 413 UV intensity (mW cm^−2^): 5 Type of reactor: fixed bed cylindrical	GC-FID HPLC	Major: Benzaldehyde; Minor (small amount): Benzene, Benzyl alcohol; Trace amounts: Benzoic acid, Phenol.	[42]
(1) Degussa P25 TiO_2_ 75% anatase/25% rutile with a BET surface area of 50 m^2^/g (2) 0.2 wt.% Pt/TiO_2_	Inlet toluene conc. (ppm): 100 Reaction temperature: room temperature UV intensity (mW cm^−2^): 2.5 λ(max), UV: 356 nm	GS-MS	Humidified and un-humidified toluene: Major: CO_2_; Small amount: Benzene, Benzaldehyde.	[50]
PC500 TiO_2_ 100% anatase BET specific surface area: 300 m^2^ g^−1^ Crystal size: 5–10 nm.	Inlet toluene conc. (ppbv): 20–400 Flow rate (mL min^−1^): 70–350 Reaction temperature (K): 298 ± 2 UV intensity (mW cm^−2^): 4.3 Type of reactor: annular flow -through	ATD-GC-MS	0% RH Major: CO_2_; (Small amount): Benzaldehyde, Benzene; Minor (Trace amount): Formaldehyde, methyl glyoxal, Vinyl methyl ketone.	[48]
			40% RH Major: CO_2_; (Small amount): Benzaldehyde; Minor (Small amount): (o,m,p-)-cresol, Benzene, Benzyl alcohol, Phenol.	
Degussa P25	Inlet toluene conc. (ppbv): 450; 1200; 8000; 3200 Flow rate (l min^−1^): 0.55 Reaction temperature (°C): 24.0–26.0 UV intensity (mW cm^−2^): 0.43–0.95 λ (UV): 254 nm Contact time: 0.2 s. Type of reactor: plate-type UV-PCO	PRT MS GS-MS	47–50% RH Formaldehyde, Methanol, Propylene, Acetaldehyde, Formic acid /ethanol, Acetone/propionaldehyde, Acetic acid, Benzene, Benzaldehyde, Benzyl alcohol, Phenol, -methyl-.	[55]
Degussa P25 TiO_2_	Inlet toluene conc. (ppbv): 1–1000 UV intensity (mW cm^−2^): 10 ± 1 λ (UV): 365 nm Type of reactor: batch Pyrex	TDS-GS/MC/FID HPLC/UV FTIR	Major: CO_2_; Minor: Aromatics: Benzene, Phenol, Benzaldehyde, Cresols; Aldehydes: Formaldehyde, Acetaldehyde, Acroleine, Pentanal, Heptanal; Others: 2-methylfurane.	[56]
Pt/TiO_2_ BET specific surface area: 67 m^2^ g^−1^ Crystal size: 5–10 nm.	Inlet toluene conc. (ppm): 1000 Weight hourly space velocity (mL g^−1^ h^−1^): 40,000 Reaction temperature (°C): 120–210 UV intensity (mW cm^−2^): Type of reactor: fixed-bed quartz tube	GS-MS	<160 °C Benzene, Nonbornane, o-xylen, p-xylen, Benzaldehyde, Phthalic acid; >160 °C Acetone, Acetic acid, Maleic anhydride, Itaconic anhydride, Benzene, Nonbornane, o-xylen, p-xylen, Benzaldehyde, Phtalic acid.	[57]

Recently, the photocatalytic removal of toluene over Pt/TiO_2_ as a function of reaction temperature was investigated [57]. The authors showed that at a temperature lower than 160 °C and the relatively high inlet toluene concentration at 1000 ppm, new species in gas phase, such as nonbornane, o-xylene, p-xylene, and phthalic acid, were detected. Additionally, they reported reaction product species with less than six carbon atoms only at temperatures higher than 160 °C, suggesting that the ring opening could only occur at temperatures above 160 °C.

### 5.2. Catalyst-Bound By-Products

Investigation of adsorbed species on the catalyst surface using solid–liquid extraction can be helpful to identify the decomposition mechanisms of aromatic molecules in addition to compounds detected in the gas phase. By-products are extracted from the catalyst with different solvents, usually with the catalyst treated after steady-state conditions. As no studies have shown that the identified species were not formed during the extraction, interpretation may be limited. We should mention the study performed by Larson et al., which can be considered a pioneering one on adsorbed species produced during TPO and TPD [52]. In this study, the authors suggested that intermediates are much more strongly bound to TiO_2_ than the original aromatics and that these intermediates are not aldehydes or alcohols formed by reaction with the methyl groups. They indicated that benzaldehyde, benzyl alcohol, and cresol are unlikely to be less-reactive intermediates forming during toluene PCO, as these compounds can be thermally decomposed during TPD or TPO.

In a later studies using water and methanol, the reaction products benzoic acid, benzyl alcohol, and benzaldehyde were identified in the TiO_2_ catalyst after the continuous-flow PCO of toluene [49,58]. Using in situ FT-IR spectroscopy, the authors reported the accumulation of benzoic acid on the catalyst surface, yielding catalyst deactivation [58]. The authors also identified the other minor species presented in Table 2 [49]. The results suggest that the direct hole transfer from the semiconductor to the aromatic predominates over the attack aromatic ring by a OH• radicals [49]. Since PCO of toluene was conducted under fixed relative humidity (5% RH), it is difficult to assess the effect of water vapor on the formation of certain by-products based on the results obtained by d’Hennezel et al. [49].

In a study by Sleiman et al. using an annular flow-through reactor, the impact of relative humidity on the reaction process was determined during continuous-flow experiments [48]. They observed that benzaldehyde, benzoic acid, and traces of benzene and formic acid could be detected at both RH level (0% and 40% RH), while cresol, benzyl alcohol, 3-hydroxybenzaldehyde, and hydroquinone were detected only at 40% RH. Furthermore, for 40% RH, hydroxylated intermediates were found to accumulate on the surface during PCO of toluene. An interesting result obtained by these groups is that, despite the partial accumulation of hydroxylated intermediates, no evidence of loss of photocatalytic activity was observed within 48 h of experiments.

Later, other authors used pulses of water vapor to evaluate the effect of various compounds on the deactivation of TiO_2_ [55]. In detail, they observed with instant increase in water vapor (under UV off) species with molecular mass of 42, 46, 60, 78, and 106, which were assigned to propylene, formic acid/ethanol, acetic acid, benzene, and benzaldehyde, respectively. Trace concentrations of species with molecular masses of 54, 56, 72, 86, and 122 (identified as acrylaldehyde, butyraldehyde, pentanal, benzoic acid, and salicylaldehyde, respectively) were desorbed under the action of water vapor from the surface of the catalyst. Hence, the authors concluded that these species are formed under the lack of water vapor, are strongly adsorbed to TiO_2_ surface, and were responsible for the deactivation of the photocatalyst. It is worth noting that most of the compounds observed on the catalyst surface by Mo et al. are highly hydrophilic compounds (Tables S1 and S2) [55]. Subsequently, Dhada et al. studied PCO using high toluene concentrations simulated by continuous reactor operation and identified additional species like hexane, cyclohexane, and crotonaldehyde on the catalyst surface [59].

**Table 2:** Solid-phase by-products identified during PCO over TiO_2_.

Catalyst/Method of Preparation	Experimental Conditions	Analytical Method	Solvent	Identified Gaseous Products	Ref.
Degussa P-25 TiO_2_ Average particle size of about 21 nm and a specific surface area of around 50 m^2^/g 8% SiO_2_-TiO_2_	Inlet toluene conc. (ppm): 30–200 Reaction temperature (K): 623 Type of reactor: in situ FTIR cell continuous flow	GS-MS GC-MS/DS	Methanol	Benzaldehyde, Benzyl alcohol, Benzoic acid.	[58]
Degussa P25, BET specific surface area: 50 m^2^ g^−1^	Inlet toluene conc. (ppmv): 13.1 Flow rate (cm^3^ s^−1^): 1 or 2 UV intensity (mW cm^−2^): λ(max), UV: 365 nm Type of reactor: continuous flow	GC-MS HPLC	Diethyl ether Water	Major: Benzoic acid, Benzyl alcohol, Benzaldehyde; Minor: 4-hydroxybenzoic acid, 4-hydroxybenzyl alcohol, 4-hydroxybenzaldehyde, and 3-hydroxybenzaldehyde Formic and acetic acids; A peak at 35 min perhaps corresponded to muconic acid (2,4-hexadienedioic acid).	[49]
PC500 TiO_2_ 100% anatase BET specific surface area: 300 m^2^ g^−1^ Crystal size: 5–10 nm.	Inlet toluene conc. (ppbv): 20–400 Flow rate (ml min^−1^): 70–350 Reaction temperature (K): 298 ± 2 UV intensity (mW cm^−2^): 4.3 Type of reactor: annular flow-through	GC-MS HPLC-UV Ion-chromatography	Solvent mixture of methanol/water (20/80 *v*/*v*)	0% RH Major: Benzaldehyde, Benzoic acid; Minor (Trace amount): Benzene, Formic acid.	[48]
				40% RH Major: Benzaldehyde, Benzoic acid; Minor (Trace amount): Benzene, Formic acid; Additional: Cresols, Benzyl alcohol, 3-hydroxybenzaldehyde, Hydroquinone.	
Degussa P25	Inlet toluene conc. (ppbv): 450; 1200; 8000; 3200 Flow rate (l min^−1^): 0.55 Reaction temperature (C): 24.0–26.0 UV intensity (mW cm^−2^): 0.43–0.95 λ (UV): 254 nm Contact time: 0.2 s Type of reactor: plate-type UV-PCO	PRT MS GS-MS	Instant concentration pulse water	Butadiene, Acrylaldehyde, Butyraldehyde, Pentanal, Butyrolactone, Benzoic acid, Salicylaldehyde.	[55]
TiO_2-x_N_x_ powder samples BET specific surface area: 67 m^2^ g^−1^	Inlet toluene conc. (ppm): 20 UV intensity (mW cm^−2^): 4.3 λ > 420 nm Type of reactor: IR-cell	IR spectrometer GC-MS IC (ICS-2000, Dionex Corporation, Sunnyvale, CA, USA) equipped with a conductivity detector	Water Ether	Major: Oxalic acid, Acetic acid, Formic acid, Pyruvic acid; In the early stage of PCO: Propionic acid, Isovaleric acid, Succinic acid.	[60]
Nano-sized TiO_2_ with a size of 5−10 nm	Inlet toluene conc. (ppm): 206 UV intensity (mW cm^−2^): 0.95–3.1 Type of reactor: IR-cell	In situ DRIFTS On-line mass-spectrometer		Major: Benzaldehyde, Benzoic acid; Minor: Benzyl alcohol.	[61]
Activated carbon fibers (ACFs)-supported TiO_2_ photocatalystTiO_2_/ACF Degussa P-25: surface area 50 m^2^/g, non- porous, about 80% anatase	λ (UV): 254 nm Constant temperatures (*T*, 25 ± 0.5 °C) Type of reactor: A Stainless-steel environmental condition-controlled chamber	GC-MS GC-FID	Carbon disulfide (CS_2_)	Major: Benzaldehyde, Benzyl alcohol; Minor: Benzoic acid, 2-methyl, p-benzoquinone, Cresol.	[62]
TiO_2_ using sol gel method	Inlet toluene conc. (mg/m^3^): 170 UV: UV-C λ (UV): 254 nm Temperature: 22.4 ± 2.3 Type of reactor: batch reactor, mimicking the continuous operation of reactor	GC-MS	Methanol	Major: Acetone; Minor: Hexane, 1,4-benzoquinone, Benzaldehyde.	[59]

Over the past decade, in situ diffuse reflectance infrared Fourier-transform spectroscopy (DRIFTS) has been used to study surface species formed during PCO [10,61]. Usually, in situ DRIFTS is carried out in combination with GC analysis to confirm the identity of products. The use of DRIFTS allows to measure online evolution of species formation during toluene oxidation and to identify the features of decomposition and accumulation of intermediate products. Briefly, DRIFTS experiments are conducted in a high-temperature cell with an IR- and UV-transparent window and includes the following main stages: (1) removal of pollutants from the cell and from catalyst surface by heating and purging with pure carrier gases, (2) adsorption of pollutants (usually at ppm level) on the catalyst surface until saturation, (3) performing photocatalytic oxidation (usually in close chamber), and (4) catalyst regeneration and monitoring of gases desorption by heating and purge with appropriate gases. With this in mind, in situ DRIFTS can be considered as a transient-type experiment, as the process of surface oxidation of a toluene-saturated catalyst proceeds in a closed chamber. In 2005, Irokawa et al. studied the photodegradation of toluene over nitrogen-doped TiO_2_ under visible-light irradiation using DRIFT in combination with GC and IC [60]. They reported the formation of various carboxylic acids and benzaldehyde in the early stage of the photo-oxidation.

## 6. PCO-Reaction Pathways of Toluene

The structure of toluene consists of two types of carbon and hydrogen atoms in the methyl group and in the aromatic ring. Thus, oxidation of toluene can take place on the methyl group or on the aromatic ring. In addition, toluene ring opening to form small mass species is one of main routes of its photocatalytic oxidation.

### 6.1. Methyl Group Oxidation

Numerous reports show that during the initial stages of the PCO reaction, benzaldehyde, benzyl alcohol, and benzoic acid are the main species formed on the catalyst surface [58,63]. The small amounts of benzene and benzyl alcohol identified at the initial stage of PCO in most of the studies indicate that these species do not play an important role in the catalyst deactivation. Thus, it may be concluded that benzene and benzyl alcohol reaction pathways are likely suppressed and that these pathways are not rate-limiting steps toward toluene photocatalytic oxidation.

**Figure 3:**
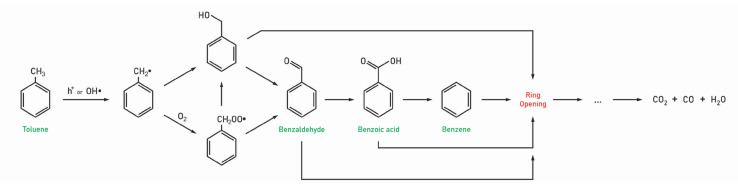
Possible PCO pathways of toluene toward methyl group oxidation.

The formation of these aromatics occurs through the benzyl radical, which is further oxidized into the peroxybenzyl radical. Coronado and Soria were able to experimentally observe electron-spin resonance (ESR) signals, which were assigned to the formation of the benzylperoxy radical [64]. They also stated that the formation of benzyl radical is favored with other possible reaction products because delocalization of the unpaired electron in the aromatic ring provides additional stability to these species. Possible reaction pathways are presented (Figure 3) for the formation of the aromatic compounds based on this literature review. In this and subsequent schemes, for simplicity, we neglect the protons, water, and other molecules for balanced reactions.

Even if there was a unanimous opinion on the surface species formed during PCO, the compounds responsible for the catalyst deactivation are still under debate. Specifically, benzoic acid has been suggested to be responsible for the deactivation of TiO_2_ because it is strongly adsorbed at the TiO_2_ surface [46,58,63]. Cao et al. used IR and TPO experiments to confirm that the deactivation of catalysts arises from the irreversible adsorption of reaction intermediates such as benzaldehyde and benzoic acid [63]. Furthermore, IR results reveal that benzoic acid is more difficult to oxidize on TiO_2_ surfaces than benzaldehyde, leading to the catalyst deactivation. They also stated that the formation of the carbonyl group makes the benzyl ring even more inert because the conjugation of the carbonyl group reduces the electron density on the benzyl ring.

Other groups suggest that benzaldehyde is more likely to accumulate and is therefore responsible for the catalyst deactivation [54,62]. For example, T. Guo et al. observed that benzaldehyde was the most abundant compound among all intermediates accumulated on the photocatalyst under all RH [62]. This is because benzaldehyde is more difficult to oxidize than benzoic acid, and the step from benzaldehyde to benzoic acid should be the slowest step during the PCO process of toluene.

The existing conflicting information may be due to different inlet concentrations of toluene used in PCO studies as well as using different experimental set-ups and different experimental modes (transient or continuous flow). With regard to toluene concentration, Sleiman et al. pointed out that inlet pollutant concentration could drastically affect the intermediate products’ accumulation and thus the catalyst deactivation. They determined a low amount of accumulated species on the surface compared to results described in previous studies in which toluene concentrations were at least a hundred times higher than used in another study [48]. As for different experimental designs [50], transient studies are ideal for investigating photocatalytic reactions; however, care must be taken when comparing results obtained between transient and continuous flow. Specifically, during transient PCO, only a monolayer of organic molecules reacts, while with continuous-flow condition, gas-phase organic molecules can replenish the adsorption site, followed by significant changes of the reaction rate.

It is worth noting that, depending on the experimental conditions, the formation of both benzaldehyde and benzoic acid is possible. For example, Z. Chen et al. studied the mechanism of the photocatalytic toluene degradation using XPS, in situ DRIFTS, and on-line MS and found that both benzaldehyde and benzoic acid are the main products accumulated, and they occupied the active site on the TiO_2_ surface [61]. One explanation for both benzaldehyde and benzoic acid accumulation is based on the reaction energy: benzaldehyde and benzoic acid have high reaction energy for ring opening on TiO_2_ among the other aromatic species presented in Table 1; as a result, the accumulation of benzaldehyde and benzoic acid occurs on the TiO_2_ surface. Prediction of processes by reaction energy values should be used with care to describe the oxidation process because the formation rate is influenced by the activation energy rather than the reaction energy. Furthermore, benzaldehyde and benzoic acids are more hydrophilic than toluene based on their dipole moments (3.0 D for benzaldehyde and 1.72 for benzoic acid vs. 0.375 D for toluene) (see Supplementary Table S1 for detail).

### 6.2. Aromatic Ring Oxidation

Hydroxylated intermediates like cresols or phenol are generally observed in gas phase, while other hydroxylated species are identified in the solid phase during toluene PCO (see Table 1 and Table 2), which suggests that reactive oxygen species, especially OH• radicals, could attack the aromatic ring, leading to its oxidation. Aromatic ring oxidation was considered as a minor PCO reaction path of toluene because typically only trace concentrations of hydroxylated by-products are reported. However, this reaction pathway may contribute to toluene PCO by formation of water and increasing RH. Specifically, the authors identified the accumulation of the aromatic ring oxidation by-products with the increase in RH. Sleiman et al. showed that hydroxylated products are relatively slowly degraded and are likely responsible for the drop of toluene mineralization when RH increased [48]. It should be noted that the formation of cresol is undesirable due to its difficult further oxidation by TiO_2_ [52], which may lead to accumulation and catalyst deactivation.

**Figure 4:**
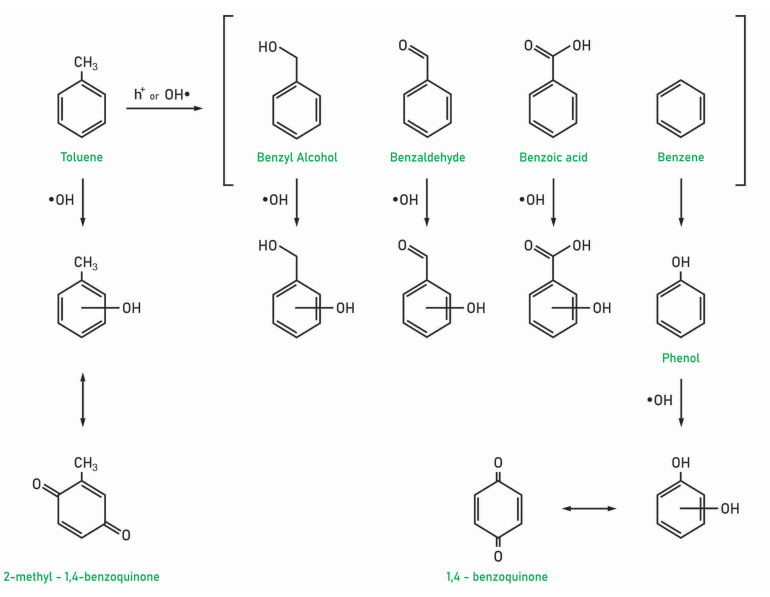
Possible PCO pathways of toluene toward aromatic ring oxidation.

Possible pathways of hydroxylated byproducts formation are presented in Figure 4. Using quantum chemistry, Frankcombe and Smith investigated the toluene oxidation reaction pathway and determined that ketone intermediates are likely formed during the reaction (toluene oxides) to cresols [15]. Other authors observed the formation of ketones such as 2-methyl-1,4-benzoquinone at trace concentrations [61,62].

### 6.3. Ring Opening

**Figure 5:**
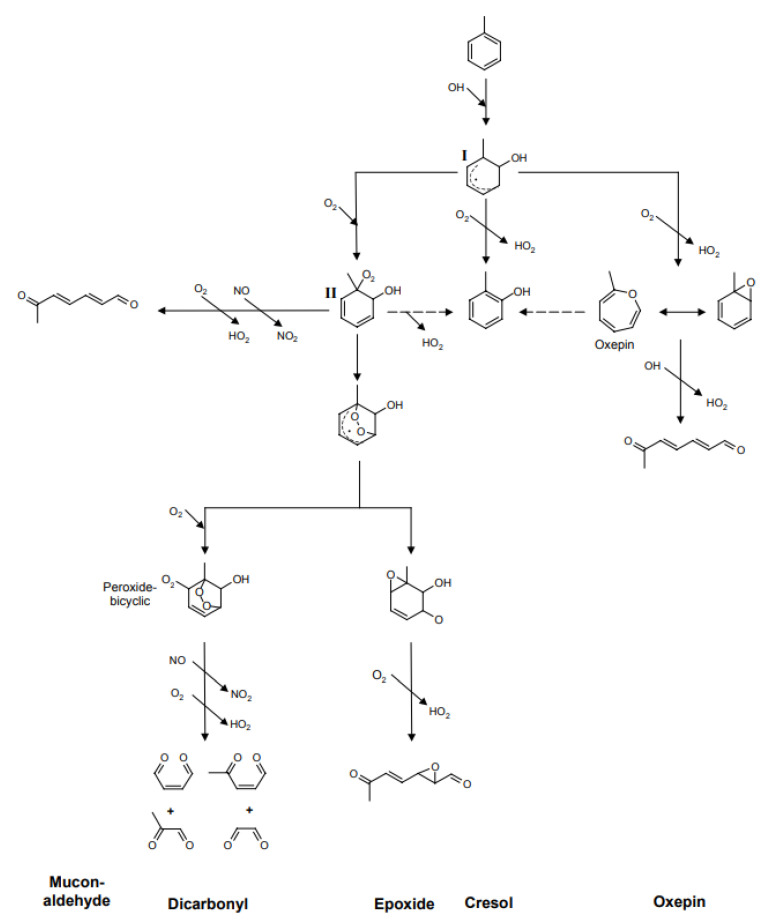
Initial reaction steps of the OH initiated oxidation of toluene as discussed in current literature. Figure by Wagner et al. [17] under a CC BY-NC-SA 2.5 license.

**Figure 6:**
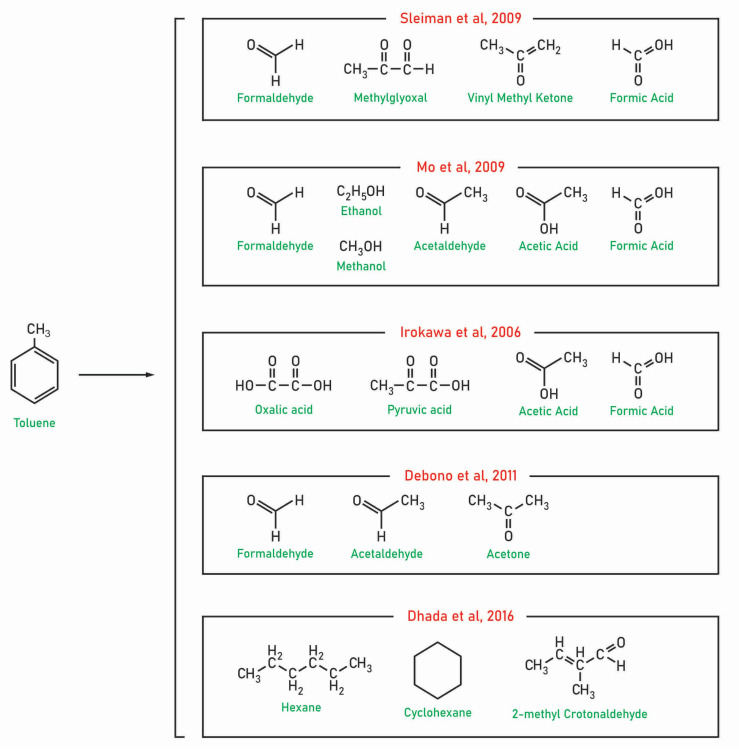
Identified toluene degradation by-products before full mineralization [48,55,56,59,60].

Detection of lower-molecular-weight species in gas and solid phases than aromatic derivatives points to the ring-opening evidence. If the initial step of the photocatalytic oxidation with ring-retaining pathways is relatively well understood, the ring-opening mechanism is speculative. The current literature review on the ring-opening aspect can be summarized as follows: 

At present, no detailed study of ring-cleavage mechanisms during PCO of toluene can be identified, and studies on toluene ring opening during PCO generally refer to the homogeneous photo-oxidation mechanism [15,57,65,66]. This photo-oxidation mechanism of toluene proceeds through the three reaction paths presented in Figure 5. These routes can be labeled as dicarbonyl route (glyoxal and methylglyoxal as the ring-opening product), epoxide route (epoxides—dominant reaction products), and an aromatic oxide/oxepin route (oxide/oxepin as reaction intermediates) [17]. These lower-molecular-weight reaction products are typically not identified in heterogeneous photocatalytic oxidation (see Table 2 or Figure 6). Thus, if photocatalytic oxidation of toluene occurs along these pathways, further oxidation of these species by ROS or direct hole transfer leading to the formation of simple carboxylic, aldehyde, and ketone species is likely to occur. In Figure 6, an oxidation scheme showing the detected ring-opening products before full mineralization is provided.

Reactive oxygen species formed on the TiO_2_ surface during UV irradiation (see reaction Equations (3)–(11)) play an important role in toluene oxidation. Coronado and Soria investigated toluene oxidation at the molecular level by electron-spin resonance spectroscopy and identified several oxygenated radicals like O^•−^, O_2_^•−^, and O_3_^•−^ [64]. Recently, using ESR via DMPO capturing, Chen et al. showed the generation of OH• and HO_2_• radicals under UV light irradiation of P25 TiO_2_ [10]. Most studies indicate that among active species, the OH• radicals seem the most important species in aromatic ring-cleavage processes, which involve a ring-opening mechanism. Other studies showed that small species were also found at RH = 0, and the authors suggested that OH• radicals are not the main active species during toluene PCO [48,49]. Taking into account that valence band holes are powerful oxidants (+2.83 V vs. NHE for anatase [24]), then direct hole oxidation is very likely.

Ring opening is generally considered to be the rate-limiting step for overall toluene decomposition [11,67]. Li et al., by calculating the OH•-driven reaction coordinates by the NEB method, found that different activation energies on selected catalysts (BiOCl) are required for the ring-opening reaction at different ring-containing intermediates [11]. The highest energy barrier was reported for toluene and the lowest for benzoic acid. The authors stated that the ring-opening reaction plays a key role in determining the overall decomposition efficiency, which can be significantly enhanced once benzyl becomes fully oxidized to benzoic acid.

## 7. Risk Assessment of By-Products

Table 3 shows the intermediates/byproducts in the gas phase identified in various works, resulting from the PCO of toluene as well as the exposure limits recommended by the National Institute for Occupational Safety and Health [68] and the Occupational Safety and Health Administration [69]. Among the compounds detected in gas phase, only benzene and formaldehyde belong to Group 1 as carcinogenic to humans and acrolein to Group 2A as probably carcinogenic to humans, in accordance with the International Agency for Research on Cancer (IARC) Monographs [70]. The U.S. Environmental Protection Agency (EPA) has included benzene under its A grouping (human carcinogen), while acetaldehyde and formaldehyde are under its B1 and B2 groups as probable human carcinogens. Among the identified by-products (benzene, acetaldehyde, methanol, propionaldehyde, acrolein, and toluene), the Reference Concentration for Inhalation Exposure (RfC) is available under the EPA Integrated Risk Information System [71]. The RfC is defined as an estimate (with uncertainty spanning perhaps an order of magnitude) of a continuous inhalation exposure to the human population (including sensitive subgroups) that is likely to be without an appreciable risk of deleterious effects during a lifetime.

Mo et al. calculated the HRI index, defined as the ratio of concentration of pollutant in gas phase to recommended exposure limit [55]. They observed that the HRI index of outlet was always higher than that of the inlet gas composition. Furthermore, their calculation showed that for inlet toluene concentration at 8 ppm, the HRI value for outlet exceeded 1. In contrast, the risk assessment through estimations of hazard index and cancerous risk in the order of 10^−6^ and overall HI is well below 1 [59]. 

This review suggests that the overall risk for formation and exposure to hazardous intermediates is low during PCO of toluene. At the same time, the generation of even small amounts of compounds with strict permissible exposure limits (see, for example, benzene, formaldehyde, or acrolein) leads to a significant deterioration in air quality indices. It is difficult to judge the effectiveness of photocatalytic oxidation for air purification because toluene in these experiments was a model pollutant that was subjected to mineralization for CO_2_ and H_2_O with different efficiencies. From the Table, we can see that toluene RfC at 5 mg/m^3^ is the highest among other by-products (see, for example, RfC of acetaldehyde 9 × 10^−3^). It is assumed that such compounds with strict regulatory levels will also be subject to mineralization during the photocatalytic air purification.

## 8. Perspectives and Conclusions

Purification by photocatalytic oxidation of volatile organics is a promising technology for air treatment owing to its low energy requirements and cost, especially if solar energy is used. Like any other treatment method, photocatalytic oxidation purification technology has a number of limitations for practical applications. Among them is deactivation of the catalyst due to the generation of strongly bound intermediates, which block active sites of the catalyst. Furthermore, generated by-products may be more toxic than the native aromatics, including the formation of cancerogenic compounds.

This review shows that in terms of fundamental aspects (mechanism of photocatalytic oxidation), attention should be paid to the following:

- Better experimental measurement and identification of intermediate organic radicals (benzyl, peroxybenzyl, etc.) formed as a result of the catalyst illumination. To date, published research has only indirectly identified peroxybenzyl radical formation by assigning ESR signals;
- Consideration of those species that play a key role in the oxidation of toluene, as there is a significant body of literature indicating that photo-oxidation may take place either by OH• attack or by direct hole transfer. At the same time, the role of O_2_**^·^**^−^ radicals is still not clear in the PCO of organic compounds in the gas phase, although the reduction of the adsorbed oxygen with surface-trapped electrons can be the rate-determining step.

Also, theoretical calculations of adsorption energy, reaction energy, and energy barriers to identify rate-limiting reactions should be performed as well as investigation of reaction kinetics. At present, ring fragmentation is considered a rate-determining step, while ring opening can, as shown by this review survey, proceed via different pathways depending on the type of accumulated aromatic species on the catalyst surface.

We recommend investigating the influence of other organic and inorganic molecules on the photo-oxidation mechanism of the target pollutant. For example, Mahmood et al. showed a lower degradation efficiency of toluene (52%) in a mixture with benzene and p-xylene than in isolated mode (100%) [43]. In addition, there are still few works on the mechanisms of photodegradation of aromatic compounds upon irradiation with visible light. Other recommendations for further improvements for photocatalytic oxidation of volatile organic pollutants can be summarized as follows:

- Development of deactivation-resistant catalysts that promote photocatalytic efficiency during progressive organic compound degradation by reducing accumulations of strongly bound and less-reactive intermediates on the catalyst surface. As an example, Li et al. used a facet-tailoring strategy on BiOCl to promote the selective ring opening at the benzoic acid intermediate [11];
- By means of modulation of the dopant coordination configuration and electron geometry in borocarbonitride, the lone electrons of carbon transform into delocalized counterparts, so it is possible to directly attack the aromatic ring, facilitating the degradation of toluene [67].

Finally, further study of the potential for reactive intermediates’ exposure and toxicity during various PCO modes with a focus on risk assessment using inhalation unit risk factors will contribute to a better understanding of photocatalytic oxidation performance. It is important to minimize the formation of hazardous compounds with strict permissible exposure limits, often having carcinogenic effects. The compounds of particular concern are benzene, formaldehyde, and acrolein. Monitoring exposure during the PCO should involve hazard indexes like maximum hazard quotient (MHQ) and the total hazard index (THI) and be helpful to determine the PCO purification efficiency (see, for example, [72]). Studies to assess air quality improvement under realistic conditions (BTEX concentrations at ppb level, UV/VIS light, and continuous-flow mode) with multicomponent composition must be conducted; in particular, a detailed assessment of reactor/reaction variables affecting by-product formation is necessary.

## Figures and Tables

**Table 3:** Carcinogenic classification and exposure limits for the identified intermediates/byproducts in the gas phase.

Compounds	^1^ CAS No.	IARC Carcinogenic Classification [70]	^2^ REL, NIOSH [68]	Reference Concentration for Inhalation Exposure (RfC) mg/m^3^ [71]	^3^ OSHA PEL [69]
Formaldehyde	50-00-0	Group 1, Carcinogenic to humans	Ca TWA 0.016 ppm C 0.1 ppm (15 min)	B1 (Probable human carcinogen—based on limited evidence of carcinogenicity in humans), Guidelines for Carcinogen Risk Assessment (U.S. EPA, 1986)	TWA 0.75 ppm ST 2 ppm
Benzene	71-43-2	Group 1, Carcinogenic to humans	Ca TWA 0.1 ppm ST 1 ppm	3 × 10^−2^ A (Human carcinogen), Guidelines for Carcinogen Risk Assessment (U.S. EPA, 1986)	TWA 1 ppm ST 5 ppm
Acrolein	107-02-8	2A, Probably carcinogenic to humans	TWA 0.1 ppm (0.25 mg/m3) ST 0.3 ppm (0.8 mg/m3)	2 × 10^−5^	TWA 0.1 ppm (0.25 mg/m^3^)
Acetaldehyde	75-07-0	Group 2B, Possibly carcinogenic to humans	Ca	9 × 10^−3^ B2 (Probable human carcinogen—based on sufficient evidence of carcinogenicity in animals), Guidelines for Carcinogen Risk Assessment (U.S. EPA, 1986)	TWA 200 ppm (360 mg/m³)
Maleic anhydride	108-31-6	-	TWA 1 mg/m^3^ (0.25 ppm)		TWA 1 mg/m^3^ (0.25 ppm)
m-Cresol p-Cresol o-Cresol	108-39-4 106-44-5 95-48-7	-	TWA 2.3 ppm (10 mg/m^3^)		TWA 5 ppm (22 mg/m^3^) (skin)
Formic acid	64-18-6		TWA 5 ppm		TWA 5 ppm
Phenol	108-95-2	Group 3, Not classifiable as to its carcinogenicity to humans	TWA 5 ppm (19 mg/m^3^) C 15.6 ppm (60 mg/m^3^) (15 min)	-	TWA 5 ppm (19 mg/m^3^)
Acetic acid	64-19-7	-	TWA 10 ppm (25 mg/m^3^) ST 15 ppm (37 mg/m^3^)		TWA 10 ppm (25 mg/m^3^)
Pentanal	110-62-3		TWA 50 ppm (175 mg/m^3^)		-
o-xylen p-xylen	95-47-6 106-42-3	Group 3, Not classifiable as to its carcinogenicity to humans	TWA 100 ppm (435 mg/m^3^) ST 150 ppm (655 mg/m^3^)		TWA 100 ppm (435 mg/m^3^)
Toluene	108-88-3		TWA 100 ppm (375 mg/m^3^) ST 150 ppm (560 mg/m^3^)	5	TWA 200 ppm C 300 ppm 500 ppm (10 min maximum peak)
Methanol	67-56-1		TWA 200 ppm (260 mg/m^3^) ST 250 ppm (325 mg/m^3^) (skin)	2 × 10^1^	TWA 200 ppm (260 mg/m^3^)
Acetone	67-64-1		TWA 250 ppm (590 mg/m^3^)	-	TWA 1000 ppm (2400 mg/m^3^)
Ethanol	64-17-5		TWA 1000 ppm		TWA 1000 ppm
Propionaldehyde	123-38-6			8 × 10^−3^	
methyl glyoxal	78-98-8	Group 3, Not classifiable as to its carcinogenicity to humans	-	-	-
Propylene	115-07-1	Group 3, Not classifiable as to its carcinogenicity to humans			
Benzaldehyde	100-52-7	-	-	-	-
Benzyl alcohol	100-51-6	-	-	-	-
Benzoic acid	65-85-0	-	-	-	-
Vinyl methyl ketone	78-94-4	-	-	-	-
Nonbornane	279-23-2				
phthalic acid	88-99-3	-			
Itaconic anhydride	2170-03-8	-	-	-	-
Pentanal	110-62-3		TWA 50 ppm (175 mg/m^3^)		-
Heptanal	111-71-7				
2-methylfurane	534-22-5				

**Note: ^1^** CAS No. Index of Chemical Abstracts Service Registry Numbers; **^2^** the NIOSH recommended exposure limits (REL s) are listed first in this section. For NIOSH RELs, “TWA” indicates a time-weighted average concentration for up to a 10 h workday during a 40 h workweek. A short-term exposure limit (STEL) is designated by “ST” preceding the value; unless noted otherwise, the STEL is a 15 min TWA exposure that should not be exceeded at any time during a workday. A ceiling REL is designated by “C” preceding the value; unless noted otherwise, the ceiling value should not be exceeded at any time. Any substance that NIOSH considers to be a potential occupational carcinogen is designated by the notation “Ca”. **^3^** The OSHA-permissible exposure limits (PEL s) and TWA concentrations for OSHA PEL s must not be exceeded during any 8 h work shift of a 40 h workweek. A STEL is designated by “ST” preceding the value and is measured over a 15 min period unless noted otherwise. OSHA ceiling concentrations (designated by “C” preceding the value) must not be exceeded during any part of the workday; if instantaneous monitoring is not feasible, the ceiling must be assessed as a 15 min TWA exposure.

## Data Availability

Some data are presented in the Supplementary Materials. Other data are available from the authors if needed for review.

## Supplementary Materials

The following supporting information can be downloaded at: https://www.mdpi.com/article/10.3390/molecules28186451/s1, Table S1. Some thermodynamic parameters of identified Gas-phase by-products during PCO over TiO_2_ [73]. Table S2. Some thermodynamic parameters of identified solid-phase by-products during PCO over TiO_2_ [73].
