# Supplementary material for: Reaction Steps in Heterogeneous Photocatalytic Oxidation of Toluene in Gas Phase—A Review
Source: Molecules. 2023 Sep 6;28(18):6451. doi: 10.3390/molecules28186451 (PMC10536914; doi:10.3390/molecules28186451)
Supplement: Supplementary file 1 [file molecules-28-06451-s001.zip › molecules-2567530-supplementary.pdf]

Supplemental Materials

Table 1S. Some thermodynamic parameters of identified Gas-phase by-products during PCO over TiO_2[i]

Compounds	CAS No.	Dipole moment μ/D	Boiling temperature, °C	$\Delta_f H^o$, kJ·mol^{-1}	$\Delta_f G^o$, kJ·mol^{-1}	S^o, J·mol^{-1}·K^{-1}	C_p, J·mol^{-1}·K^{-1}
Formaldehyde (g)	50-00-0	2.332(2)	-19.1(0.5)	-108.6	-102.5	218.8	35.4
Benzene (l)	71-43-2	0	80.08(0.07)	49,1	124,5	173,4	136,0
Acrolein	107-02-8	*Trans* 3.117(4)<br>*Cis* 2.552(3)	52.3(0.1)				
Acetaldehyde (l)	75-07-0	2.750(6)	20.8(0.6)	-192,2	-127,6	160,2	89,0
Maleic anhydride (g)	108-31-6	3.95[ii]	52.56(0.04)	-398,3			
m-Cresol (l)<br>p-Cresol (c)<br>o-Cresol (c)	108-39-4<br>106-44-5<br>95-48-7	1.48<br>1.48<br>1.45	202.2(0.1)<br>201.9(0.1)<br>191.0(0.1)	-194.0<br>-204.6<br>-199.3		212.6<br>165.4<br>167.3	224.9<br>154.6<br>150.2
Formic acid (l)	64-18-6	1.425(2)	101	-425.0	-361.4	129.0	99.0
Phenol (c)	108-95-2	1.224(8)	181.8(0.1)	-165.1		144.0	127.4
Acetic acid (l)	64-19-7	1.70(3)	117.9(0.2)	-484.3	-389.9	159.8	123.3
Pentanal (l)	110-62-3	2.57[ii]	103(2)	-267.2			
o-xylene (l)<br>m-xylene (l)<br>p-xylene (l)	95-47-6<br>108-38-3<br>106-42-3	0.640(5)	144.4(0.4)<br>139.1(0.4)<br>138.3(0.5)	-24.4<br>-25.4<br>-24.4			186.1<br>183.0<br>181.5
Toluene (l)	108-88-3	0.375(10)	110.60(0.07)	12.4		157.3	50.5
Methanol (l)	67-56-1	1.70(2)	64.5(0.7)	-239.2	-166.6	126.8	81.1
Acetone (l)	67-64-1	2.88(3)	56.08(0.07)	-248.4		199.8	126.3
Ethanol (l)	64-17-5	1.69(3)	78.24(0.09)	-277.6	-174.8	160.7	112.3
Propionaldehyde (l)	123-38-6	2.72	48.0(0.2)	-215.6			
methyl glyoxal (l)	78-98-8		72(15)	-309.1			
Propylene (g)	115-07-1	0.366(1)	-47.6(0.1)	20.0			
Benzaldehyde (l)	100-52-7	3.0	178.7(0.4)	-87.0		221.2	172.0

Benzyl alcohol (l)	100-51-6	1.71(9)	205.3(0.2)	-160.7		216.7	217.9
Benzoic acid (c)	65-85-0	1.72 [ii]	250.2(0.6)	-385.2		167.6	146.8
Vinyl methyl ketone	78-94-4	2.90 ± 1.08 [ii]	81(4)				
Nonbornane (c)	279-23-2		107(6)	-95.1			151.0
phthalic acid (c)	88-99-3	2.60 [ii]	150	-782.0		207.9	188.1
Itaconic anhydride	2170-03-8						
Pentanal (l)	110-62-3	2.57 [ii]	103(2)	-267.2			
Heptanal (l)	111-71-7	2.56 [ii]	153	-311.5		335.4	230.1
2-methylfuran (l)	534-22-5	0.65(5)	63.9(0.2)				

Table 2S. Some thermodynamic parameters of identified solid-phase by-products during PCO over TiO_2[iii]

Compounds	CAS No.	Dipole moment, μ/D	Boiling temperature, °C	$\Delta_f H^o$, kJ·mol^{-1}	$\Delta_f G^o$, kJ·mol^{-1}	S^o, J·mol^{-1}·K^{-1}	C_p, J·mol^{-1}·K^{-1}
4-hydroxybenzoic acid	99-96-7	2.76[ii]					
4-hydroxybenzyl alcohol	623-05-2		252				
4-hydroxybenzaldehyde	123-08-0	4.22[ii]					
3-hydroxybenzaldehyde	100-83-4	2.78[ii]	240				
muconic acid (2,4-hexadienedioic acid)	**1119-72-8**	0.36[ii]	228				
p-Hydroquinone (c)	123-31-9	2.38(5)	288(5)	-364.5			136.0
1,3-Butadiene (l)	106-99-0	0[ii]	-4.6(0.2)	88.5		199.0	123.6
Butyraldehyde (l)	123-72-8	2.72[ii]	74.8(0.2)	-239.2		246.6	163.7
γ-Butyrolactone (l)	96-48-0	4.27(3)	204.6(0.4)	-420.9			141.4
Salicylaldehyde (l)	90-02-8	2.86	208(5)				222.0
Oxalic acid (c)	144-62-7		157				
Pyruvic acid	127-17-3	2.30[ii]	165	-829.9		109.8	91.0
Propionic acid	79-09-4	1.75(9)	141.5(0.2)	-510.7		191.0	152.8
Isovaleric acid (l)	503-74-2	0.63	176.5(0.2)	-561.6			
Succinic acid (c)	110-15-6		234(3)	-940.5		167.3	153.1
p-benzoquinone (c)	106-51-4	0.66[ii]	sub	-185.7			129.0
Hexane (l)	110-54-3	0.08[ii]	68.72(0.06)	-198.7			195.6

[i] Haynes, W.M. (Ed.). (2016). CRC Handbook of Chemistry and Physics (97th ed.). CRC Press. https://doi.org/10.1201/9781315380476

[ii] http://www.stenutz.eu/chem/dipole.php

[iii] Haynes, W.M. (Ed.). (2016). CRC Handbook of Chemistry and Physics (97th ed.). CRC Press. https://doi.org/10.1201/9781315380476
